# People who living with HIV/AIDS also have a high prevalence of anxiety disorders: a systematic review and meta-analysis

**DOI:** 10.3389/fpsyt.2024.1259290

**Published:** 2024-02-06

**Authors:** Jiahao Ji, Yang Zhang, Yundong Ma, Lin Jia, Miaotian Cai, Zhen Li, Tong Zhang, Caiping Guo

**Affiliations:** ^1^ Center for Infectious Diseases, Beijing Youan Hospital, Capital Medical University, Beijing, China; ^2^ Beijing Institute of Sexually Transmitted Disease Prevention and Control, Beijing, China; ^3^ Beijing Key Laboratory of Mental Disorders, National Clinical Research Center for Mental Disorders & National Center for Mental Disorders, Beijing Anding Hospital, Capital Medical University, Beijing, China; ^4^ Advanced Innovation Center for Human Brain Protection, Capital Medical University, Beijing, China; ^5^ Department of Respiratory and Critical Care Medicine, Beijing Youan Hospital, Capital Medical University, Beijing, China

**Keywords:** anxiety disorders, human immunodeficiency virus (HIV), acquired immunodeficiency syndrome (AIDS), people living with HIV/AIDS, systematic review, meta-analysis

## Abstract

**Background:**

An estimated 301 million people worldwide suffer from anxiety disorders. People living with HIV/AIDS (PLWHA) are particularly prone to anxiety disorders that could interfere with the important developmental process in an individual’s development and ultimately result in a wide range of negative mental, physical, and psychosocial consequences, as well as poor quality of life in those population groups. Early intervention for anxiety disorders can reverse some of the physical damage caused by anxiety. However, based on systematic reviews and meta-analyses, the specific prevalence of anxiety disorders in PLWHA remains unknown.

**Method:**

We conducted a literature search on PubMed, Embase, and Web of Science up to 22 October 2022. A random-effects meta-analysis was used to pool prevalence rates from the included studies. Sensitivity and subgroup analyses were performed to identify the possible sources of heterogeneity and to compare the prevalence estimates across groups. The Joanna Briggs Institute’s Quality Assessment Checklist was used to assess the quality of the included studies. Cochran’s Q and I^2^ tests were used to assess the between-study heterogeneity.

**Results:**

Ten studies with a total of 238,570 cases were included for the final analysis. Results showed that 15.5% of HIV/AIDS patients had anxiety disorders. The prevalence was higher in females (20.8%) than males (20.7%). The mean age of PLWHA with anxiety disorders was 46.58 ± 11.15 years in these included studies. The subgroup analyses showed significant higher prevalence in non-heterosexual (32.1%).

**Conclusion:**

We attempted to quantify literature that could allow for stronger inferences to be made regarding the significantly higher prevalence of anxiety disorders in PLWHA, a finding that suggests the imperativeness of intervention strategies to alleviate suffering and reduce the probable negative ramifications.

**Systematic review registration:**

https://www.crd.york.ac.uk/prospero/display_record.php?ID=CRD42023442219, identifier CRD42023442219.

## 1 Introduction

There were about 39.0 (33.1 - 45.7) million people living with human immunodeficiency virus (HIV)/acquired immunodeficiency syndrome (AIDS) (PLWHA) globally in 2022 (1). While much progress has been made in the care of HIV/AIDS patients, a variety of challenges still lie ahead. The intersectionality of HIV/AIDS is rooted in the structural and environmental factors such as underdevelopment and poverty, instability of legal and policy environments, and social stigma that have negatively impacted the physical and mental health of PLWHA, especially among identified key populations, including men who have sex with men, prisoners, people who inject drugs, sex workers and transgender people (2).

The availability of antiretroviral therapy (ART) has transformed HIV/AIDS into a chronic disease that is treatable but presently difficult to cure completely (3). With the prolonged survival of PLWHA, HIV-associated neurocognitive and psychiatric disorders (such as anxiety disorders, depressive disorders, etc.) will not only affect the choice of ART regimens and patient compliance but also may lead to treatment failure (4), which has. Comorbidities have become a major contributor to the quality of survival and prognosis of HIV-infected patients.

There are many potential crisis points in dealing with HIV, from the initial diagnosis to co-morbidities, daily strict medication intake, hospitalization, permanent disability, disfigurement and stigma. For PLWHA, having a psychiatric condition often means worse treatment adherence and outcomes (5, 6).

There are a variety psychiatric disorders associated with HIV/AIDS. Disorders including depression, psychosis, substance use disorder, and post-traumatic stress disorder are 1.5 to 8 times higher among PLWHA (6). As for PLWHA, anxiety disorders are one of the most common psychiatric illnesses in HIV-associated mental disorders (7, 8). Overall, different studies have reported the incidence of anxiety disorders among people living with HIV/AIDS ranging from 0.6% to 68.2% (9–18). In the general population, anxiety is a risk factor for cognitive impairment (19), it can adversely affect the brain (20), and also it has a negative impact on disorders other than those related to the neuropsychiatric system (21). Several studies have reported higher suicidality among PLWHA (22, 23), and anxiety is related to greater suicidality (24). Anxiety disorders affect the prognosis of HIV, delay the time to viral suppression, and increase the rate of antiretroviral failure even after suppression (25). Due to self and other discrimination caused by illness, as well as negative psychological factors such as pessimism and depression, PLWHA’s quality of life is relatively decreased (26).

The relationship between anxiety disorders and HIV/AIDS remains uncertain. An investigation constructed a heuristic model, which demonstrated that the correlation between HIV/AIDS and anxiety symptoms and disorders is both reciprocal and dynamic. It was found that HIV/AIDS and anxiety can mutually intensify one another through various distinct mechanisms (5). Based on the available studies, what we can know is that for PLWHA, due to their specificity, the experience of HIV infection may be associated with anxiety psychopathology (6, 27). On the other hand, anxiety disorders, as non-HIV-related adverse events, can also affect ART regimen selection and treatment compliance, lead to treatment failure, and severely disrupt the survival and quality of life of HIV-infected patients. Anxiety may be involved in behaviors that increase the risk of HIV infection, such as unprotected sex and substance use (28), and increases the risk of suicide in HIV-infected patients (24). Due to the lack of in-depth involvement of neurology and psychiatry specialists, HIV-related psychiatric disorders such as anxiety disorders are poorly treated, and there is a complex diagnostic process that requires prevention, treatment and health promotion for these conditions.

Anxiety disorders are common among mental disorders in PLWHA, previous studies have indicated that the median rate of anxiety disorders, as determined through questionnaire-based assessments, was 33.3% (5). Nevertheless, it is important to note that these studies relied on self-rating scales rather than employing more stringent diagnostic criteria, which may introduce limitations in terms of diagnostic accuracy. Self-report questionnaires, such as the Brief Symptom Inventory (BSI) (29), and the Generalized Anxiety Disorder 7-Item Scale (GAD-7) (30) are commonly employed for screening anxiety symptoms and disorders. A previous systematic review found that diagnostic interviews resulted in significantly higher rates of diagnosis of anxiety disorders compared to questionnaire-based assessments (5). Although these self-rated scales offer convenience and expeditious evaluation of the individual’s psychological condition, the reliability of the diagnostic outcomes remains a subject of debate. The current clinical diagnosis of anxiety disorders is based primarily on the Diagnostic and Statistical Manual of Mental Disorders (DSM) (31) and the International Classification of Diseases (ICD) (32). In the case of PLWHA, the diagnosis and treatment of anxiety disorders require the involvement of both infectiologists and psychiatrists. Consequently, in this study, we strictly adhered to the DSM or ICD diagnostic criteria for anxiety disorders when screening articles to ensure diagnostic accuracy. The DSM-5 and ICD-11 defined anxiety disorders covered in the current review are shown in **Table 1** (DSM-5) and **Table 2** (ICD-11).

**Table 1:** DSM-5 defined anxiety disorders.

Anxiety disorders	Marked symptoms
Generalized anxiety disorder	• Excessive anxiety or worry about many events or activities (anxious anticipation)
Separation anxiety disorder	• Excessive fear and anxiety about leaving home or being separated from attachment figures
Selective mutism	• Inability to start speaking when meeting other individuals in social interactions, or to respond when spoken to
Specific phobia	• Fear or anxiety when there is a specific situation or object named as the source of the phobic stimulus
Social anxiety disorder	• Significant or excessive fear or anxiety about social situations in which the individual may be judged by others
Panic disorder	• Recurrent and unexpected panic attacks
Agoraphobia	• Significant or excessive fear or anxiety triggered by real or anticipated exposure to different situations

**Table 2:** ICD-11 defined anxiety disorders.

ICD-11 Code	Title	Definition
6B00	Generalized anxiety disorder	marked symptoms of anxiety that persist for at least several months, for more days than not
6B01	Panic disorder	recurrent unexpected panic attacks that are not restricted to particular stimuli or situations
6B02	Agoraphobia	marked and excessive fear or anxiety that occurs in response to multiple situations where escape might be difficult or help might not be available
6B03	Specific phobia	a marked and excessive fear or anxiety that consistently occurs when exposed to one or more specific objects or situations and that is out of proportion to actual danger
6B04	Social anxiety disorder	marked and excessive fear or anxiety that consistently occurs in one or more social situations
6B05	Separation anxiety disorder	marked and excessive fear or anxiety about separation from specific attachment figures
6B06	Selective mutism	consistent selectivity in speaking
6B0Y	Other specified anxiety or fear-related disorders	/
6B0Z	Anxiety or fear-related disorders, unspecified	/

In the present study, we aim to quantify several research questions by applying meta-analysis, including: What is the prevalence of anxiety disorders in PLWHA? Do anxiety disorders experience equal or higher prevalences among PLWHA than among HIV-free individuals? Are there any risk factors for anxiety disorders in PLWHA? In addition, we assessed the variability in effect size estimates for potential moderation by age, gender, marital status, educational status, employment status, and duration of HIV infection for potential moderation. Although we would like to assess the impact of factors such as duration of antiviral therapy, type of antiviral drug, history of mental disorders, geographic and racial/ethnic variation, and different social disciplines, etc., the current studies provided us with insufficient information for further analysis. This knowledge informs the need for further development and evaluation of interventions for PLWHA that address anxiety disorders. By approaching the above questions, we provide clearer guidance on how anxiety disorders in PLWHA can be alleviated through social, psychological and biological interventions.

## 2 Method

This protocol has been registered on PROSPERO with registration number CRD42023442219. (https://www.crd.york.ac.uk/PROSPERO/display_record.php?RecordID=442219).

### 2.1 Information sources and search strategy

Electronic databases from PubMed, Web of Science, and EMBASE were searched to select relevant publications through October 22, 2022. Search terms included: Web of Science: (TS = (HIV) OR TS = (Human Immunodeficiency Virus) OR TS = (AIDS) OR TS = (PLWH)) AND (TS = (anxiety disorder) OR TS = (anxiety)); PubMed: (“HIV” OR “Human Immunodeficiency Virus” OR “AIDS” OR “PLWH”) AND (“anxiety disorder” OR “anxiety”); Embase: (“HIV” OR “Human Immunodeficiency Virus” OR “AIDS” OR “PLWH”) AND (“anxiety disorder” OR\ “anxiety”).

### 2.2 Selection process and eligibility criteria

Two investigators (J.J. and Z.Y.) independently assessed the literature included in the review. Any disagreements were discussed and a consensus was reached. Discrepancies were resolved through discussions between J.J. and Z.Y. to achieve consensus. Studies were included if they satisfied the criteria of (1) being published in English in peer-reviewed journals; (2) including both cross-sectional and extracting baseline data from prospective studies; (3) providing the proof of HIV infection and status; (4) complying with diagnostic criteria for anxiety disorders based on the DSM or the ICD; (5) providing adequate data (including the number of participants in both groups) in order to calculate an effect size. Exclusion criteria included: (1) studies in non-English languages; (2) review papers as well as full papers that were not available; (3) no valid diagnostic measure of anxiety disorders or a symptomatological measure of anxiety disorders. Eligibility criteria were applied during two steps: (1) title and abstract screening; (2) full-text screening.

### 2.3 Data collection process

The data was extracted with pre-tested structured forms. In addition to bibliographic data, the extraction process sought the following information: population, study design, sample size, gender, age, outcome measures, and key outcomes.

### 2.4 Quality assessment

The Joanna Briggs Institute quality assessment tool was utilized to estimate the quality of studies included in the final analysis. Studies were scored on a frequency scale, with responses of yes, no, unclear, and not applicable. We used the total number of positive scores to perform the calculation of the total quality score for each study.

In the present study, all statistical analyses were performed using the comprehensive meta-analysis software version 3 (33). Prevalence rates were pooled across studies by a random-effects meta-analysis (34). I^2^ statistics were used to assess heterogeneity between studies (34). I^2^ statistics values of 75%, 50%, and 25% respectively represent high, medium, and low heterogeneity (35). Participants’ gender, marital status, educational status, employment status, duration of HIV infection, duration of ART treatment, and CD4 count were used to assess potential sources of heterogeneity across studies. Egger’s regression test and funnel plots were used to weigh the risk of publication bias. The significance level α was set at 0.05 for all analyses.

## 3 Results

**Figure 1:**
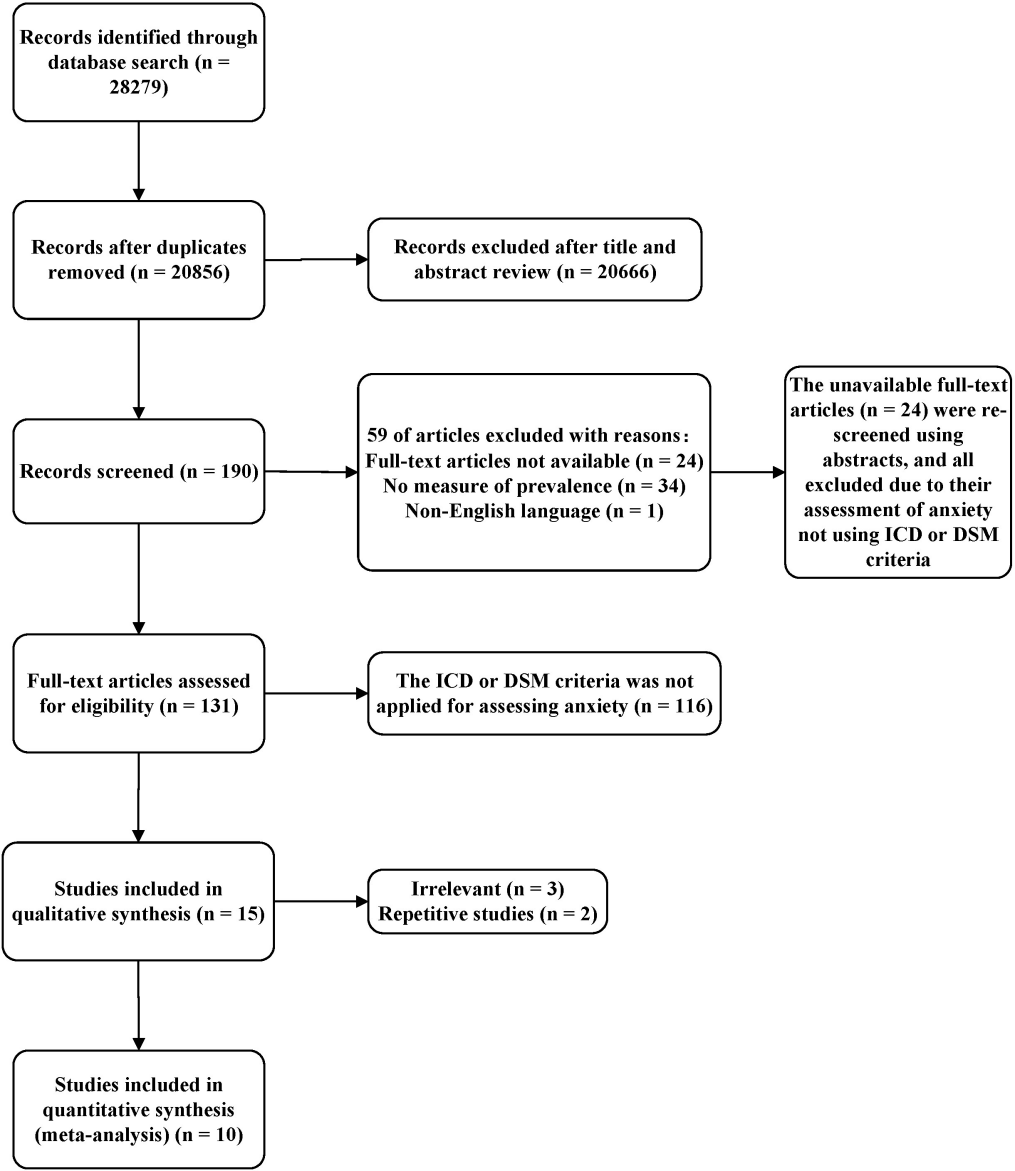
PRISMA flow chart of study selection.

The PRISMA flow chart (see **Figure 1**) illustrates how studies were selected in this review and meta-analysis. Our search strategy yielded an initial total of 28,279 articles. In total, 20,856 potentially relevant articles remained after duplicates were removed. The titles and abstracts of these articles were then screened and 20,638 articles were excluded. A total of 218 full-length articles were subsequently reviewed. Finally, 10 articles that met both the inclusion and exclusion criteria were included. Each of these 10 articles reported clinically diagnosed anxiety disorders by DSM or ICD (see **Tables 1**, **2**).

### 3.1 Study characteristics

Key characteristics of the studies included in this systematic review and meta-analysis are shown in **Table 3**. A total of 10 articles (9–18) with 238,570 participants living with HIV/AIDS, published between 2012 and 2022, were included in the final analysis. Half of the included studies were performed in the United States (50%; n = 5) and the other half were conducted in undeveloped countries including Africa (n = 3), China (n = 1) and Malaysia (n = 1). Sample sizes for the included articles ranged from 300 participants in Lagos, Nigeria to 122,896 participants in the United States. Two instruments were used to assess participants for anxiety disorders: DSM (6 studies) and ICD (4 studies).

**Table 3:** The characteristics of studies included in the systematic review and meta-analysis.

Study name	Event(n)	Total(n)	Prevalence (%)	Method	Country/Area
Olagunju 2012 (9)	65	300	21.7	ICD-10	Lagos, Nigeria
Parhami 2013 (10)	1264	7834	16.1	ICD-9	California. USA
Van Den Heuvel 2013 (11)	285	418	68.2	M.I.N.I.(DSM-IV)	Zambia
Leyro 2015 (12)	64	139	46.0	DSM-IV Axis I Disorders non-Participant Edition	San Francisco, USA
Levy 2019 (13)	708	5904	12.0	DSM-V	Washington DC, USA
Brown 2020 (14)	225	37438	0.6	ICD-9	USA
Lang 2022 (15)	34219	122896	27.8	ICD-9/ICD-10	USA
Liu 2022 (16)	2016	63000	3.2	DSM-V	China
Ong 2022 (17)	82	191	42.9	M.I.N.I.(DSM-III)	Malaya
Wanjala 2022 (18)	24	450	5.3	DSM-IV	Kenya

### 3.2 Quality of included studies

**Table 4** presents the quality of the studies included in this review. Seven studies (70%) had an adequate sample size to determine the prevalence of anxiety disorders. All studies (100%) utilized standard instruments or valid diagnostic criteria to measure anxiety disorders and suitable statistical analysis to explore the prevalence of anxiety disorders. The articles involved in the final analysis had a mean quality score of 6.90, ranging from 6 to 9, based on the Joanna Briggs Institute Quality Evaluation Checklist. Six studies (60%) were high-quality studies (scored above 6.9) and the remaining were fair-quality articles (scored between 6 and 6.9) (**Table 4**).

**Table 4:** Qualities of studies included in the systematic review and meta-analysis.

Study name	Response									
	Q1	Q2	Q3	Q4	Q5	Q6	Q7	Q8	Q9	Total
Olagunju 2012 (9)	Y	Y	N	Y	Y	Y	Y	Y	U	7
Parhami 2013 (10)	Y	Y	Y	Y	Y	Y	Y	Y	U	8
Van Den Heuvel 2013 (11)	N	N	Y	Y	Y	Y	Y	Y	U	6
Leyro 2015 (12)	N	Y	Y	N	Y	Y	Y	Y	U	6
Levy 2019 (13)	N	Y	Y	Y	Y	Y	Y	Y	U	7
Brown 2020 (14)	Y	Y	Y	Y	Y	Y	Y	Y	U	8
Lang 2022 (15)	Y	Y	Y	Y	Y	Y	Y	Y	U	8
Liu 2022 (16)	N	Y	Y	N	Y	Y	Y	Y	U	6
Ong 2022 (17)	Y	Y	N	Y	Y	Y	Y	Y	U	7
Wanjala 2022 (18)	Y	Y	N	N	Y	Y	Y	Y	U	6

Keys

Q1–Q9 represent questions used to assess the quality of included studies, which are listed below.

Q1: Was the sample frame appropriate to address the target population?

Q2: Were study participants sampled in an appropriate way?

Q3: Was the sample size adequate?

Q4: Were the study subjects and the setting described in detail?

Q5: Was the data analysis conducted with sufficient coverage of the identified sample?

Q6: Were valid methods used for the identification of the condition?

Q7: Was the condition measured in a standard, reliable way for all participants?

Q8: Was there appropriate statistical analysis?

Q9: Was the response rate adequate, and if not, was the low response rate managed appropriately?

Y, yes; N, no; U, unclear; NA, not applicable.

### 3.3 Prevalence of anxiety disorders among PLWHA

**Figure 2:**
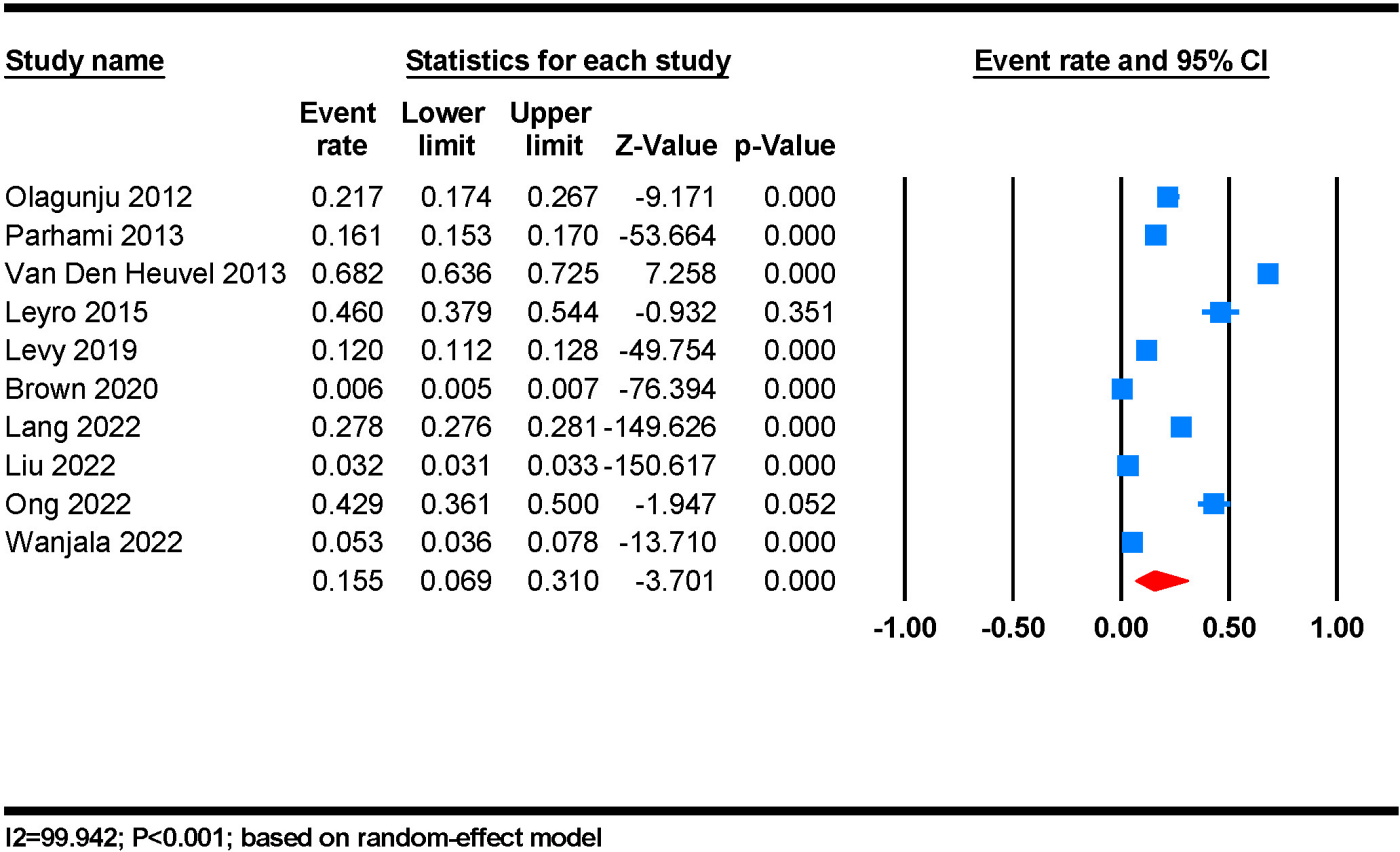
The prevalence of anxiety among people living with HIV/AIDS: a random-effect meta-analysis.

The pooled prevalence of anxiety disorders among PLWHA was found to be 15.5% (95% CI 6.9 – 31.0). There was significant heterogeneity across the studies used for this analysis (I^2 =^ 99.942%; P < 0.001) (**Figure 2**). The pooled age estimate of anxiety disorders among HIV individuals was 46.58 ± 11.15 years.

### 3.4 Subgroup analysis

A subgroup analysis of the prevalence of anxiety disorders in PLWHA was performed. Gender, marital status, educational status, employment status and duration of HIV infection were further analyzed and summarized in **Table 5**.

**Table 5:** Sensitivity analysis of all studies based on sex, marital status, educational status, and employment status.

Groups		Number of studies (n)	Sample size	Combined Prevalence Rate (%)	95%CI	P value	I²	*P* value of group difference
Sex	Male	3	111139	20.7	12.5-32.3	< 0.001	99.596	>0.05
	Female	3	19755	20.8	16.1-26.5	< 0.001	89.610	
Sexual orientation	Heterosexual	3	26275	21.7	14.3-31.5	< 0.001	96.801	0.006
	Non-heterosexual	3	104646	32.1	27.1-37.5	< 0.001	97.648	
Marital status	Single	2	5109	20.1	11.9-31.9	< 0.001	80.241	>0.05
	Married	2	581	14.1	11.5-17.2	< 0.001	0.000	
	Divorced/Widows/Others	2	178	21.1	11.2-36.3	0.001	75.953	
Educational status	Primary and Secondary	3	4035	21.3	13.9-31.1	< 0.001	88.151	>0.05
	Tertiary and Post-Tertiary	3	3132	28.5	12.9-51.7	0.068	97.145	
Employment status	Employed	2	3233	27.6	6.6-67.2	0.261	98.939	>0.05
	Unemployed	2	4448	22.0	12.4-35.8	< 0.001	80.828	

#### 3.4.1 Sociodemographic factors

In relation to sexual orientation, non-heterosexual (32.1%) among PLWHA exhibited a significant higher prevalence of anxiety disorders when compared to heterosexual (21.7%) (P = 0.006). Concerning gender, three studies provided available data for analysis, with a higher prevalence of anxiety disorders in females (20.8%) than in males (20.7%), although this result is not significant (**Table 5**). In our subgroup analysis of marital status, a total of 2 studies were included, we found that the prevalence of anxiety disorders was high for participants who were divorced or widows 21.1% (95% CI 11.2 – 36.3) followed by single 20.1% (11.9 - 31.9) and married 14.1% (95% CI 11.5 - 17.2), likewise, the observed difference was not statistically significant (P > 0.05) (**Table 5**). Moreover, in the subgroup of educational status, prevalence can be calculated in 3 studies. We found that among individuals who received tertiary or post-tertiary education, anxiety disorders were more common (28.5%) than among those with primary or secondary education (21.3%) (**Table 5**), though this result is insignificant (P > 0.05). For employment status, 2 studies were included for analysis. Employed HIV-infected people (27.6%) were more likely to have anxiety disorders (P = 0.707), compared with unemployed ones (22.0%), though there was no significant difference. Our study aimed to additionally include subgroup analyses for age stratification, family support, race/ethnicity, religion, medical insurance and social pressures. However, the limited availability of data prevents us from conducting such analysis. Nevertheless, the mean age values were calculated based on the available data, yielding a result of 46.58 ± 11.15 years.

#### 3.4.2 Disease-related factors

Due to limited data, we did not perform subgroup analyses for CD4 counts and duration of HIV infection. However, we calculated the mean values for these subgroups with the available data. The mean duration of HIV infection was 5.89 ± 5.94 years, meanwhile, the mean CD4 count was 446.92 ± 280.60 per mm^3^. Although we tended to find whether initial/recent CD4 count, initial HIV RNA copies (positive or negative), duration of ART, initial/recent type of ART, and self/family history of mental disorders are potential factors for anxiety disorders in PLWHA, there was little amount of data for our analysis.

### 3.5 Sensitivity analysis

**Figure 3:**
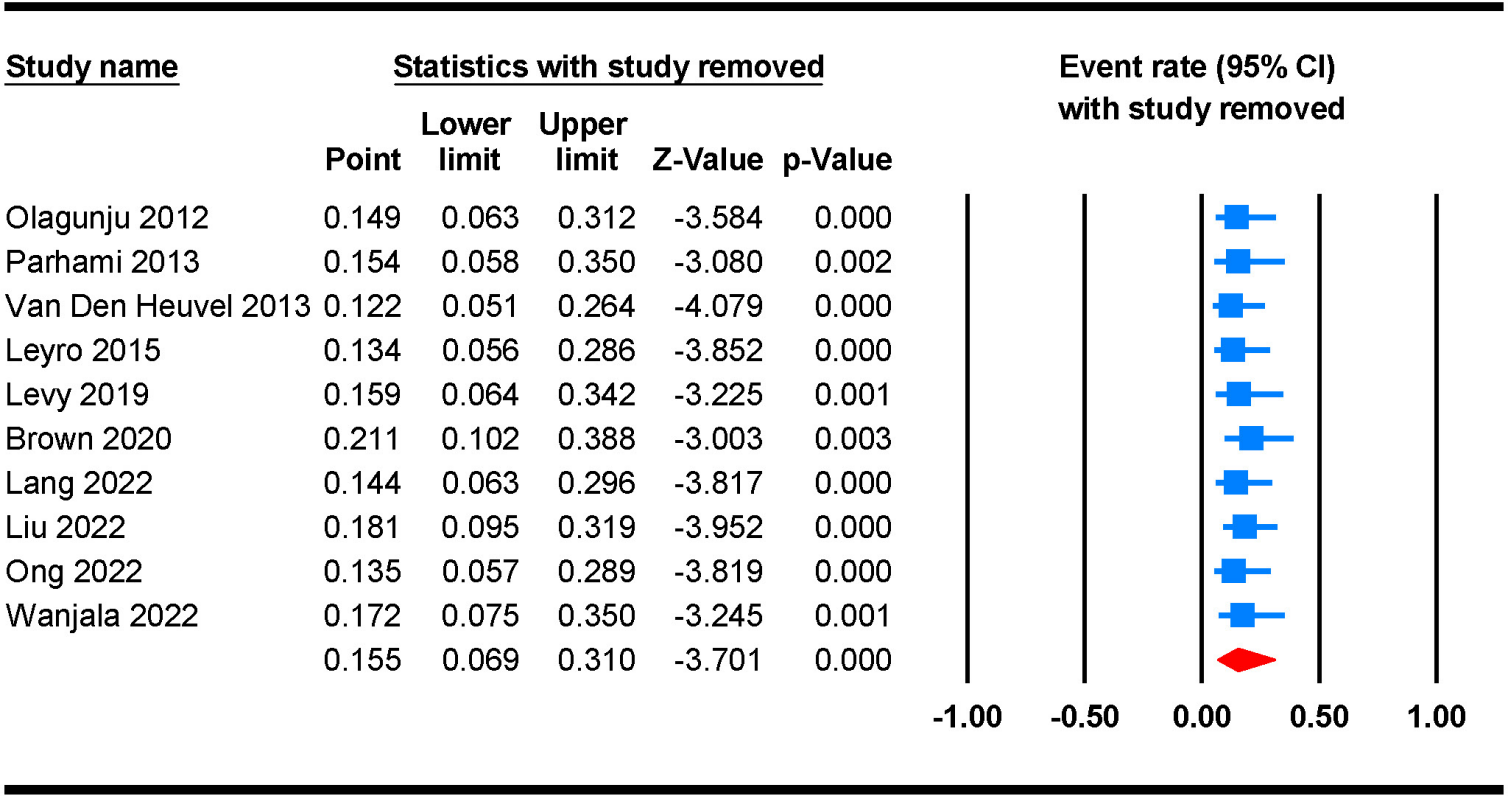
Leave-one-out sensitivity analysis for the prevalence of anxiety disorders among PLWH.

We further performed a leave-one-out sensitivity analysis to check possible causes of heterogeneity across the studies involved in the analysis. This analysis indicates that the results of the main analysis are robust and not reliant on a single study. The estimated prevalence of anxiety disorders ranged between 12.2% (95% CI 5.1 - 26.4) and 21.1 (95% CI 10.2 - 38.8) after the deletion of a single study (**Figure 3**).

### 3.6 Publication bias

**Figure 4:**
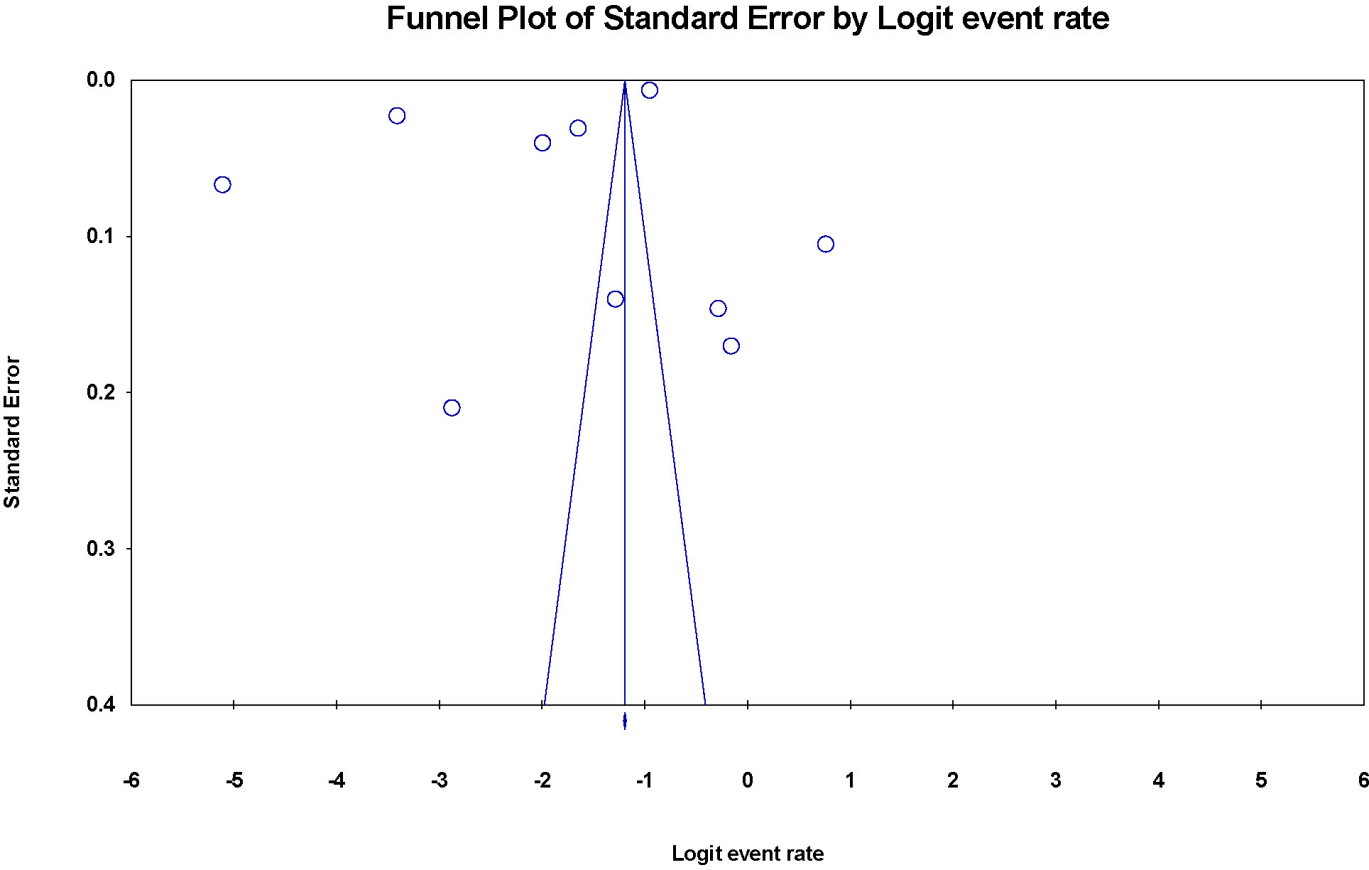
Funnel plot of the risk of publication bias for the prevalence of anxiety disorders among PLWH.

The meta-analysis demonstrated significantly different levels of heterogeneity between studies with reported prevalence of anxiety disorders (I^2 =^ 99.942%; P < 0.001). A random-effects model was used for the analysis due to the high heterogeneity. The funnel plots (**Figure 4**) indicated a potential tendency toward reporting positive findings. The fundamental causes of the asymmetry include (1) coincidence due to the relatively small number of included studies; (2) heterogeneity between studies, as in differences in methodology, including setting, and instruments for outcome measurement.

## 4 Discussion

### 4.1 Main findings

We designed this study to explore some of the issues associated with the higher prevalence of anxiety disorders in PLWHA with strict DSM or ICD diagnoses compared to those without HIV infection.

Overall, our final meta-analysis revealed that a remarkably higher proportion of PLWHA had anxiety disorders (15.5%). PLWHA with non-heterosexual sexual orientation demonstrated a significantly higher prevalence of anxiety disorders. The estimated prevalence was approximately equal for females (20.8%) and males (20.7%) in PLWHA. In addition, the mean age of anxiety disorders in PLWHA recorded in these included studies was 46.58 ± 11.15 years. The significantly higher prevalence compared to the general population suggests that anxiety disorders are an important global public health concern among PLWHA and that they require urgent attention in terms of prevention and treatment.

### 4.2 Comparisons with the existing evidence

The total estimated number of people living with anxiety disorders worldwide is 264 million, and the proportion of the global population living with anxiety disorders in 2015 was estimated to be 3.6% (36). According to a global health estimation from WHO, 301 million people worldwide suffered from anxiety disorders in 2019, including 58 million children and adolescents (37). As a result of the COVID-19 pandemic, the number of people suffering from anxiety dramatically increased in 2020. Preliminary estimates suggest that the number of people suffering from anxiety and major depression increased by 26% each in just one year. The estimated prevalence of anxiety disorders among PLWHA in the current study is 4.31 times higher than the reported prevalence of anxiety disorders in the general population. Several explanations for the higher prevalence of anxiety disorders among HIV-infected individuals than in the general population will be analyzed in the following aspects: (1) As with the stigmatization of patients with other infectious or chronic diseases in common, there is a significant shift in interpersonal, social relationships, and social roles of HIV patients that may put the HIV population at higher risk for anxiety disorders than the general population. (2) Chronic damage from other disease-related complications and poor disease prognosis may also cause disease-related stress in HIV-infected patients, leading to an increased incidence of anxiety disorders (3) The particular immune function status of HIV-infected people may lead to a higher chance of anxiety disorders in PLWHA. As demonstrated in previous studies, a marked effect of HIV infection on immunity (reduced CD4 count) and the subsequent increased risk of anxiety disorders in PLWHA with reduced CD4 count (38). A cross-sectional study suggests that opportunistic infections (co-infection with tuberculosis) in HIV-infected patients may increase the vulnerability to anxiety in HIV-infected patients (39). (4) A meta-analysis suggested that perceived discrimination has a notable negative impact on both psychological and physical health, and people who experience chronic discrimination are vulnerable to mental health, including anxiety (40). Compared to the general population, PLWHA is at greater risk of experiencing stigma, discrimination, prejudice, social association, or marginalization as a special population (41, 42), which may also be related to the greater risk of anxiety among PLWHA (43, 44).

Anxiety disorders are characterized by excessive fear and worry associated with behavioral disturbances, symptoms are significant enough to cause marked distress and severe impairment in functioning (32). Our study shows that the prevalence of anxiety disorders is a little bit higher in females than in males, which is consistent with the reported prevalence of anxiety disorders in individuals in the general population (36, 45). Possible explanations for gender differences in the prevalence of anxiety disorders include cultural, psychological, and biological factors (45, 46). Specifically, the relevance between anxiety disorders and gender differences in brain structure and function, stress response, reproductive hormone exposure, social expectation, and experience, needs to be considered (45). Recent studies with rodents have indicated gender differences in circuits and the neurobiological processes of diseases including conflict anxiety, fear processing, arousal, social avoidance, learned helplessness, and amnesia (46). However, our findings suggest a higher but insignificant prevalence of anxiety disorders in HIV-infected females. We consider that more relevant studies with larger sample sizes are required to confirm our conclusion.

Non-heterosexuals in PLWHA may be more vulnerable to suffering from anxiety disorders. This furtherly confirms the previous findings that non-heterosexuals are more prone to mental disorders (47). Previous research has indicated that individuals identified as lesbian, gay, bisexual, and transgender, are more prone to suffer prejudice and societal pressures, rendering them more susceptible to mental health disorders compared to heterosexual individuals (47, 48).

The present systematic review and meta-analysis found substantial heterogeneity in studies identifying anxiety disorders in PLWHA. The heterogeneity revealed may be attributed to the variability in participant characteristics and the methodology of the included studies. Concerning methodological differences, the included studies differ by sample size, instruments used to estimate outcomes, sampling results, and population sources. In addition, the included studies differ by the gender and residence of the participants, and the six countries from which they were selected. In addition, there were some differences in the socioeconomic and cultural contexts of the six countries that influenced the participants’ psychological status. To clarify possible sources of heterogeneity among studies, we performed stratified analyses but observed no significant variation in the prevalence of anxiety disorders in PLWHA with different gender (male and female), marital status (single, married, and divorced/widows/others), educational status (primary/secondary and tertiary/post-tertiary), employment status (employed and unemployed) (P > 0.05).

Although we expect to further analyze whether any other risk factors affect the prevalence of anxiety disorders in PLWHA, the current study data are insufficient to implement our ideas. Pleasingly, several studies have provided us with some findings. An earlier cross-sectional study (9) especially focused on the impact of religions, family support, and history of self/family past psychiatric illness on PLWHA, the result indicated that a lack of family support is a risk factor for anxiety disorders in PLWHA, although no significant differences of religion (Christianity and Islam) or history of self/family past psychiatric illness were found. A retrospective cohort study revealed significant statistical associations between psychiatric disorders and a variety of factors, such as transgender identity, homosexual and bisexual orientations, African-American ethnicity, unemployment, income levels ranging from 101-200% of the poverty level, income exceeding 200% of the poverty level, federal/state insurance coverage, and symptomatic HIV infection.

The likelihood of anxiety disorders in PLWHA may also exhibit variations based on racial differences. The findings of a survey on HIV prevalence among men who have sex with men (MSM) indicate that black and Hispanic/Latino MSM have a higher mean HIV prevalence compared to white MSM (49). Besides, Black and Hispanic/Latino MSM face various forms of stigma (50, 51), including racism and homophobia (52, 53). Parhami’s study (10) revealed a lower prevalence of anxiety disorders among African-Americans living with HIV. There has been speculation regarding the lower identification rates of psychiatric disorders in minority populations, potentially attributed to healthcare disparities (54). While some argue that minorities have a lower susceptibility to psychiatric disorders (55), others argue that cultural factors influence both patient disclosure and clinician understanding of psychiatric disorders (56). Consequently, individuals from these communities of PLWHA may be more susceptible to anxiety disorders. Furthermore, the persistence of racial and ethnic disparities in healthcare, particularly in primary care settings, can be attributed to various factors such as reduced provision of care in settings frequented by nonwhite individuals, physician bias, resource limitations, and patient-related factors (54). These factors collectively contribute to the presence of bias in estimating the prevalence of anxiety disorders among nonwhite PLWHA.

Notably, some studies suggested a correlation between insurance status and the utilization of mental health services (57, 58), as well as the subsequent outcomes. However, it is important to consider that disparities in health insurance coverage could potentially influence an individual’s propensity to seek assistance from healthcare professionals (59), thereby introducing a potential confounding factor that may impact the results.

The co-occurrence of psychiatric morbidity, substance abuse and HIV/AIDS is a worldwide phenomenon. Previous research (60) has established a higher likelihood of anxiety disorders among drug users compared to non-users. A recent meta-analysis has further indicated an overall current pooled prevalence of substance use at 25.13% (61). Consequently, it is imperative to devote additional attention and research to ascertain whether race/ethnicity, religion, sexual orientation, prejudice, social pressure, cultural factors and substance abuse may be potential factors for anxiety disorders in PLWHA.

### 4.3 Strength and limitations

The present study has several strengths. First, this is the first systematic review and meta-analysis on the prevalence estimate of anxiety disorders among individuals with HIV/AIDS. We designed this study to address concerns about the higher prevalence of anxiety disorders in PLWHA compared to HIV-free individuals. Second, multiple subgroup analyses were conducted to assess the prevalence of anxiety disorders in PLWHA under different influencing factors and then to obtain possible risk factors for it, which are important for early detection and early intervention of anxiety disorders in PLWHA. Third, we performed subgroup and sensitivity analyses depending on sociodemographic factors and disease-related factors to detect possible bias risks.

Limitations of this systematic review and meta-analysis should be considered. First, our analysis did not incorporate studies that relied on anxiety disorder diagnoses assessed by self-rating scales. Consequently, a comprehensive comparison between the prevalence of anxiety disorders diagnosed via questionnaire-based assessment and DSM/ICD could not be conducted. To address this limitation, further analyses will be performed in the subsequent sections of our study. Since PLWHA may be more distrustful of healthcare providers, this may result in barriers to seeking medical care and treatment in this population. They may be most vulnerable to psychological disorders, but have not been included in studies. In another case, individuals possessing state or private insurance exhibited a higher propensity for availing mental health services, encompassing the utilization of medications, outpatient consultations, and residential treatments, in contrast to those lacking insurance coverage (59). Due to the presence of substantial obstacles hindering marginalized groups’ access to health care (54, 62), their likelihood of receiving a DSM/ICD diagnosis would be diminished. Therefore, our findings may not accurately reflect the true prevalence of anxiety disorders within these populations. This situation might lead to biased results as our study is not representative of the entire HIV/AIDS population. Moreover, although we expected to find more information on ethnicity, religion, culture, family history, psychiatric history, immunology, etc. for subgroups analyses, the existing literature does not provide enough information to allow us to accomplish this, thus more relevant research is needed to refine this shortcoming. Additionally, half of the included studies were conducted in the same country, so the results of this study may be underrepresented, but the other half of the studies were performed in many regions of the world, which also reflects the current state of the world; furthermore, we have included studies published in English, suggesting that potential studies in other languages may have been missed.

### 4.4 Implications and prospects

The current review has several academic and clinical implications. First, since PLWHA have a significantly higher risk of developing anxiety disorders than the general population, and anxiety disorders can prolongedly and negatively affect patients’ psychological and physical functions, it is particularly vital to clarify the biological and pathophysiological mechanisms of PLWHA with anxiety for more effective early diagnosis and intervention. Future studies are therefore needed to investigate possible reasons for the higher prevalence of anxiety disorders in individuals with HIV/AIDS compared to reported estimates in the general population. Second, since half of the literature included in this study was performed in a single country, making the results not highly representative of the actual level of the world, further studies from more countries are imperative. Third, it has been demonstrated that pathological anxiety/stress damages the brain and leads to structural degeneration and functional impairment of the hippocampus and prefrontal cortex, right of increasing the risk of developing neuropsychiatric disorders (20). However, such damage can be reversed by pharmacological and non-pharmacological interventions (20). For PLWHA with anxiety disorders, an integrated and coordinated public health-based approach to early screening and intervention is urgently needed to alleviate suffering and reduce further negative consequences. Fourth, the number of eligible articles included in the subgroup analysis in this study was limited, and some possible risk factors for anxiety disorders in PLWHA were not analyzed in the subgroup due to poor data. Therefore, more data and studies on anxiety disorders in PLWHA are needed for further analysis.

## 5 Conclusion

The prevalence of anxiety disorders is significantly higher in PLWHA, and early screening and intervention for anxiety disorders in PLWHA is beneficial and necessary, given that anxiety disorders can cause long-term but partially reversible damage to the brain.

## Author contributions

JJ: Conceptualization, Data curation, Formal analysis, Investigation, Methodology, Software, Supervision, Validation, Visualization, Writing – original draft, Writing – review & editing, Resources. YZ: Conceptualization, Data curation, Formal analysis, Funding acquisition, Investigation, Methodology, Project administration, Resources, Supervision, Validation, Writing – original draft, Writing – review & editing. YM: Conceptualization, Data curation, Formal analysis, Investigation, Methodology, Software, Supervision, Validation, Visualization, Writing – original draft, Writing – review & editing. LJ: Investigation, Methodology, Software, Supervision, Validation, Visualization, Writing – original draft. MC: Data curation, Methodology, Supervision, Validation, Writing – review & editing. ZL: Funding acquisition, Project administration, Resources, Writing – review & editing. TZ: Funding acquisition, Project administration, Resources, Writing – review & editing. CG: Conceptualization, Data curation, Formal analysis, Investigation, Methodology, Supervision, Validation, Visualization, Writing – original draft, Writing – review & editing.

## References

[1] Global Hiv & Aids Statistics — Fact Sheet . (2023). Available at: https://www.unaids.org/en/resources/fact-sheet.

[2] Algarin AB Zhou Z Cook CL Cook RL Ibañez GE . Age, sex, race, ethnicity, sexual orientation: intersectionality of marginalized-group identities and enacted hiv-related stigma among people living with hiv in Florida. AIDS Behav (2019) 23(11):2992–3001. doi: 10.1007/s10461-019-02629-y 31392442 PMC6803104

[3] Fauci AS Lane HC . Four decades of hiv/aids - much accomplished, much to do. N Engl J Med (2020) 383(1):1–4. doi: 10.1056/NEJMp1916753 32609976

[4] Akinwunmi B Buchenberger D Scherzer J Bode M Rizzini P Vecchio F . Dose-related and contextual aspects of suboptimal adherence to antiretroviral therapy among persons living with hiv in Western Europe. Eur J Public Health (2021) 31(3):567–75. doi: 10.1093/eurpub/ckaa229 PMC8277220 33462616

[5] Brandt C Zvolensky MJ Woods SP Gonzalez A Safren SA O’Cleirigh CM . Anxiety symptoms and disorders among adults living with hiv and aids: A critical review and integrative synthesis of the empirical literature. Clin Psychol Rev (2017) 51:164–84. doi: 10.1016/j.cpr.2016.11.005 PMC5195877 27939443

[6] Nedelcovych MT Manning AA Semenova S Gamaldo C Haughey NJ Slusher BS . The psychiatric impact of hiv. ACS Chem Neurosci (2017) 8(7):1432–4. doi: 10.1021/acschemneuro.7b00169 PMC5769465 28537385

[7] Myer L Stein DJ Grimsrud AT Herman A Seedat S Moomal H . Dsm-iv-defined common mental disorders: association with hiv testing, hiv-related fears, perceived risk and preventive behaviours among South African adults. S Afr Med J (2009) 99(5 Pt 2):396–402. 19588804 PMC3203648

[8] Rezaei S Ahmadi S Rahmati J Hosseinifard H Dehnad A Aryankhesal A . Global prevalence of depression in hiv/aids: A systematic review and meta-analysis. BMJ Support Palliat Care (2019) 9(4):404–12. doi: 10.1136/bmjspcare-2019-001952 31537580

[9] Olagunju AT Adeyemi JD Erinfolami AR Ogundipe OA . Factors associated with anxiety disorders among hiv-positive attendees of an hiv clinic in Lagos, Nigeria. Int J STD AIDS (2012) 23(6):389–93. doi: 10.1258/ijsa.2011.011200 22807530

[10] Parhami I Fong TW Siani A Carlotti C Khanlou H . Documentation of psychiatric disorders and related factors in a large sample population of hiv-positive patients in california. AIDS Behav (2013) 17(8):2792–801. doi: 10.1007/s10461-012-0386-8 PMC3628408 23247363

[11] Van Den Heuvel L Chishinga N Kinyanda E Weiss H Patel V Ayles H . Frequency and correlates of anxiety and mood disorders among tb- and hiv-infected Zambians. AIDS Care - psychol Socio-Medical Aspects AIDS/HIV (2013) 25(12):1527–35. doi: 10.1080/09540121.2013.793263 23668833

[12] Leyro TM Vujanovic AA Bonn-Miller MO . Examining associations between cognitive-affective vulnerability and hiv symptom severity, perceived barriers to treatment adherence, and viral load among hiv-positive adults. Int J Behav Med (2015) 22(1):139–48. doi: 10.1007/s12529-014-9404-8 24643444

[13] Levy ME Monroe AK Horberg MA Benator DA Molock S Doshi RK . Pharmacologic treatment of psychiatric disorders and time with unsuppressed hiv viral load in a clinical hiv cohort. J Acquired Immune Deficiency Syndromes (2019) 82(3):329–41. doi: 10.1097/QAI.0000000000002138 PMC6791752 31356466

[14] Brown MJ Cohen SA DeShazo JP . Psychopathology and hiv diagnosis among older adults in the United States: disparities by age, sex, and race/ethnicity. Aging Ment Health (2020) 24(10):1746–53. doi: 10.1080/13607863.2019.1636201 PMC6942639 31274001

[15] Lang R Hogan B Zhu J McArthur K Lee J Zandi P . The Prevalence of Mental Health Disorders in People with Hiv and the Effects on the Hiv Care Continuum. (London, England: AIDS) (2022). doi: 10.1101/2022.04.19.22273931 PMC9782734 36541638

[16] Liu X Wang H Zhu Z Zhang L Cao J Zhang L . Exploring bridge symptoms in hiv-positive people with comorbid depressive and anxiety disorders. BMC Psychiatry (2022) 22(1):448. doi: 10.1186/s12888-022-04088-7 35790936 PMC9254609

[17] Ong JY Yee A Amer Nordin AS Danaee M Azwa RI . The prevalence of depression, anxiety and associated factors among adults with living human immunodeficiency virus in university malaya medical centre. Int J STD AIDS (2022) 33(10):880–9. doi: 10.1177/09564624221106528 35801969

[18] Wanjala SW Nyongesa MK Mwangi P Mutua AM Luchters S Newton CRJC . Measurement characteristics and correlates of hiv-related stigma among adults living with hiv: A cross-sectional study from coastal Kenya. BMJ Open (2022) 12(2):e050709. doi: 10.1136/bmjopen-2021-050709 PMC8867337 35193904

[19] Becker E Orellana Rios CL Lahmann C Rücker G Bauer J Boeker M . Anxiety as a risk factor of Alzheimer’s disease and vascular dementia. Br J Psychiatry (2018) 213(5):654–60. doi: 10.1192/bjp.2018.173 30339108

[20] Mah L Szabuniewicz C Fiocco AJ . Can anxiety damage the brain? Curr Opin Psychiatry (2016) 29(1):56–63. doi: 10.1097/YCO.0000000000000223 26651008

[21] Fonseca-Rodrigues D Rodrigues A Martins T Pinto J Amorim D Almeida A . Correlation between pain severity and levels of anxiety and depression in osteoarthritis patients: A systematic review and meta-analysis. Rheumatol (Oxford) (2021) 61(1):53–75. doi: 10.1093/rheumatology/keab512 34152386

[22] Keiser O Spoerri A Brinkhof MWG Hasse B Gayet-Ageron A Tissot F . Suicide in hiv-infected individuals and the general population in Switzerland, 1988-2008. Am J Psychiatry (2010) 167(2):143–50. doi: 10.1176/appi.ajp.2009.09050651 20008942

[23] Robertson K Parsons TD van der Horst C Hall C . Thoughts of death and suicidal ideation in nonpsychiatric human immunodeficiency virus seropositive individuals. Death Stud (2006) 30(5):455–69. doi: 10.1080/07481180600614435 16610158

[24] Quintana-Ortiz RA Gomez MA Báez Feliciano DV Hunter-Mellado RF . Suicide attempts among puerto rican men and women with hiv/aids: A study of prevalence and risk factors. Ethn Dis (2008) 18(2 Suppl 2):S2–219-24. 18646353

[25] Pence BW Miller WC Gaynes BN Eron JJ . Psychiatric illness and virologic response in patients initiating highly active antiretroviral therapy. J Acquir Immune Defic Syndr (2007) 44(2):159–66. doi: 10.1097/QAI.0b013e31802c2f51 17146374

[26] Mokgethi NO Christofides N Machisa M Akpomiemie G Lalla-Edward S . Quality of life and associated factors among people receiving second-line anti-retroviral therapy in johannesburg, South Africa. BMC Infect Dis (2022) 22(1):456. doi: 10.1186/s12879-022-07429-9 35550020 PMC9103409

[27] Mitra P Jain A Kim K . Hiv and aids in older adults: neuropsychiatric changes. Curr Psychiatry Rep (2022) 24(9):463–8. doi: 10.1007/s11920-022-01354-z 35809165

[28] Cook JA Burke-Miller JK Steigman PJ Schwartz RM Hessol NA Milam J . Prevalence, comorbidity, and correlates of psychiatric and substance use disorders and associations with hiv risk behaviors in a multisite cohort of women living with hiv. AIDS Behav (2018) 22(10):3141–54. doi: 10.1007/s10461-018-2051-3 PMC6153984 29460130

[29] Derogatis LR . Bsi Brief Symptom Inventory: Administration, Scoring, and Procedures Manual. (Minneapolis, MN: National Computer Systems) (1993).

[30] Kroenke K Spitzer RL Williams JB Löwe B . The patient health questionnaire somatic, anxiety, and depressive symptom scales: A systematic review. Gen Hosp Psychiatry (2010) 32(4):345–59. doi: 10.1016/j.genhosppsych.2010.03.006 20633738

[31] Organization AP . Diagnostic and Statistical Manual of Mental Disorders, 5th Ed. (Virginia, United States: American Psychiatric Publishing, Inc) (2013).

[32] WHO . International Classification of Diseases, Eleventh Revision (Icd-11) (2019/2021) . Available at: https://icd.who.int/browse11.

[33] Pierce C Borenstein M Hedges LV Higgins JPT Rothstein HR . Comprehensive meta-analysis (Version 2.0) [Computer software]. (Englewood, Nj: Biostat) (2008) 11(1):188–91.

[34] Borenstein M Hedges LV Higgins JPT Rothstein HR . A basic introduction to fixed-effect and random-effects models for meta-analysis. Res Synth Methods (2010) 1(2):97–111. doi: 10.1002/jrsm.12 26061376

[35] Higgins JPT Thompson SG Deeks JJ Altman DG . Measuring inconsistency in meta-analyses. BMJ (2003) 327(7414):557–60. doi: 10.1136/bmj.327.7414.557 PMC192859 12958120

[36] Organisation WH . Depression and Other Common Mental Disorders: Global Health Estimates. Geneva: World Health Organisation (2017).

[37] Paulus DJ Brandt CP Lemaire C Zvolensky MJ . Trajectory of change in anxiety sensitivity in relation to anxiety, depression, and quality of life among persons living with hiv/aids following transdiagnostic cognitive-behavioral therapy. Cogn Behav Ther (2020) 49(2):149–63. doi: 10.1080/16506073.2019.1621929 PMC6939144 31264940

[38] Agus DF Effendy E Camellia V . Screening of anxiety and depression related cd4 count of people living with hiv/aids with anti-retroviral in medan, Indonesia. Open Access Maced J Med Sci (2019) 7(16):2590–4. doi: 10.3889/oamjms.2019.396 PMC6876798 31777611

[39] Tesfaw G Ayano G Awoke T Assefa D Birhanu Z Miheretie G . Prevalence and Correlates of Depression and Anxiety among Patients with Hiv on-Follow up at Alert Hospital, Addis Ababa, Ethiopia. BMC Psychiatry (2016) 16(1):368. doi: 10.1186/s12888-016-1037-9 27806711 PMC5094082

[40] Pascoe EA Smart Richman L . Perceived discrimination and health: A meta-analytic review. Psychol Bull (2009) 135(4):531–54. doi: 10.1037/a0016059 PMC2747726 19586161

[41] Evangeli M Wroe AL . Hiv disclosure anxiety: A systematic review and theoretical synthesis. AIDS Behav (2017) 21(1):1–11. doi: 10.1007/s10461-016-1453-3 PMC5216111 27406227

[42] Armoon B Fleury M-J Bayat A-H Fakhri Y Higgs P Moghaddam LF . Hiv related stigma associated with social support, alcohol use disorders, depression, anxiety, and suicidal ideation among people living with hiv: A systematic review and meta-analysis. Int J Ment Health Syst (2022) 16(1):17. doi: 10.1186/s13033-022-00527-w 35246211 PMC8896327

[43] Vreeman RC McCoy BM Lee S . Mental health challenges among adolescents living with hiv. J Int AIDS Soc (2017) 20(Suppl 3):21497. doi: 10.7448/IAS.20.4.21497 28530045 PMC5577712

[44] Folayan MO Oginni O Ibigbami OI Philip U Mfam NN Mbam C . Associations between mental health and hiv status among sexual minority and heterosexual adolescents in Nigeria. BMJ Glob Health (2022) 7(12):e010231. doi: 10.1136/bmjgh-2022-010231 PMC9791420 36564086

[45] Altemus M Sarvaiya N Neill Epperson C . Sex differences in anxiety and depression clinical perspectives. Front Neuroendocrinol (2014) 35(3):320–30. doi: 10.1016/j.yfrne.2014.05.004 PMC4890708 24887405

[46] Bangasser DA Cuarenta A . Sex differences in anxiety and depression: circuits and mechanisms. Nat Rev Neurosci (2021) 22(11):674–84. doi: 10.1038/s41583-021-00513-0 34545241

[47] Meyer IH . Prejudice, social stress, and mental health in lesbian, gay, and bisexual populations: conceptual issues and research evidence. Psychol Bull (2003) 129(5):674–97. doi: 10.1037/0033-2909.129.5.674 PMC2072932 12956539

[48] Russell ST Fish JN . Mental health in lesbian, gay, bisexual, and transgender (Lgbt) youth. Annu Rev Clin Psychol (2016) 12:465–87. doi: 10.1146/annurev-clinpsy-021815-093153 PMC4887282 26772206

[49] Williams LD Stall R Tempalski B Jefferson K Smith J Ibragimov U . Trajectories of and disparities in hiv prevalence among black, white, and hispanic/latino men who have sex with men in 86 large U.S. Metropolitan statistical areas, 1992-2013. Ann Epidemiol (2021) 54:52–63. doi: 10.1016/j.annepidem.2020.09.004 32950653 PMC7932556

[50] Balaji AB Bowles KE Hess KL Smith JC Paz-Bailey G . Association between enacted stigma and hiv-related risk behavior among msm, national hiv behavioral surveillance system, 2011. AIDS Behav (2017) 21(1):227–37. doi: 10.1007/s10461-016-1599-z 27830344

[51] Díaz RM Ayala G Bein E Henne J Marin BV . The impact of homophobia, poverty, and racism on the mental health of gay and bisexual latino men: findings from 3 US cities. Am J Public Health (2001) 91(6):927–32. doi: 10.2105/ajph.91.6.927 PMC1446470 11392936

[52] Wong CF Weiss G Ayala G Kipke MD . Harassment, discrimination, violence, and illicit drug use among young men who have sex with men. AIDS Educ Prev (2010) 22(4):286–98. doi: 10.1521/aeap.2010.22.4.286 PMC2962624 20707690

[53] Balsam KF Molina Y Beadnell B Simoni J Walters K . Measuring multiple minority stress: the lgbt people of color microaggressions scale. Cultural Diversity ethnic minority Psychol (2011) 17(2):163–74. doi: 10.1037/a0023244 PMC4059824 21604840

[54] Lagomasino IT Stockdale SE Miranda J . Racial-ethnic composition of provider practices and disparities in treatment of depression and anxiety, 2003-2007. Psychiatr Serv (Washington DC) (2011) 62(9):1019–25. doi: 10.1176/appi.ps.62.9.1019 PMC4805374 21885579

[55] Breslau J Kendler KS Su M Gaxiola-Aguilar S Kessler RC . Lifetime risk and persistence of psychiatric disorders across ethnic groups in the United States. psychol Med (2005) 35(3):317–27. doi: 10.1017/s0033291704003514 PMC2748985 15841868

[56] McGuire TG Miranda J . New evidence regarding racial and ethnic disparities in mental health: policy implications. Health affairs (Project Hope) (2008) 27(2):393–403. doi: 10.1377/hlthaff.27.2.393 18332495 PMC3928067

[57] Goldstein RB Rotheram-Borus MJ Johnson MO Weinhardt LS Remien RH Lightfoot M . Insurance coverage, usual source of care, and receipt of clinically indicated care for comorbid conditions among adults living with human immunodeficiency virus. Med Care (2005) 43(4):401–10. doi: 10.1097/01.mlr.0000156850.86917.f8 15778643

[58] Smith SR Kirking DM . The effect of insurance coverage changes on drug utilization in hiv disease. J Acquir Immune Defic Syndr (2001) 28(2):140–9. doi: 10.1097/00126334-200110010-00005 11588507

[59] Weaver MR Conover CJ Proescholdbell RJ Arno PS Ang A Ettner SL . Utilization of mental health and substance abuse care for people living with hiv/aids, chronic mental illness, and substance abuse disorders. J Acquir Immune Defic Syndr (2008) 47(4):449–58. doi: 10.1097/QAI.0b013e3181642244 18197121

[60] Cox BJ Norton GR Swinson RP Endler NS . Substance abuse and panic-related anxiety: A critical review. Behav Res Ther (1990) 28(5):385–93. doi: 10.1016/0005-7967(90)90157-e 2256896

[61] Junaid K Afzal S Daood M Siddiqui M . Substance abuse and mental health issues among hiv/aids patients. J Coll Physicians Surgeons–Pakistan: JCPSP (2023) 33(3):325–34. doi: 10.29271/jcpsp.2023.03.325 36945165

[62] Vela MB Erondu AI Smith NA Peek ME Woodruff JN Chin MH . Eliminating explicit and implicit biases in health care: evidence and research needs. Annu Rev Public Health (2022) 43:477–501. doi: 10.1146/annurev-publhealth-052620-103528 35020445 PMC9172268

